# The German adaptation of the Amputee Body Image Scale and the importance of psychosocial adjustment to prosthesis use

**DOI:** 10.1038/s41598-025-90737-2

**Published:** 2025-02-20

**Authors:** Robin Bekrater-Bodmann

**Affiliations:** 1 Department of Psychiatry, Psychotherapy and Psychosomatics, Uniklinik RWTH Aachen, Aachen, Germany; 2 Scientific Center for Neuropathic Pain Aachen SCNAACHEN, Uniklinik RWTH Aachen, Aachen, Germany; 3 Department of Psychosomatic Medicine and Psychotherapy, Medical Faculty Mannheim, Central Institute of Mental Health, Heidelberg University, Mannheim, Germany

**Keywords:** Amputation, Body image, Pain, Patient-reported outcome measure, Prosthesis embodiment, Rehabilitation, Psychology, Health care

## Abstract

Negative cognitions related to one’s own body, here referred to as body image disturbances (BID), are common after lower limb amputation and correlate with weak psychological functioning. The Amputee Body Image Scale (ABIS) is internationally used to assess BID in persons with lower limb amputation. However, there is no psychometrically evaluated German adaptation available. Including a sample of 191 individuals with lower limb amputation, the present study developed and psychometrically evaluated the German ABIS. Results suggest high reliability in terms of internal consistency and stability of the measure over two years. Meaningful and significant relationships to sex, amputation level, post-amputation pain, mobility, and psychopathology indicate validity of the instrument. Multivariate analyses emphasize a specific and inverse relationship between BID and psychosocial adaptation to the prosthesis including its embodiment. Focussing on ABIS items that are independent of the type of amputation or rehabilitation experiences resulted in an ABIS short form with psychometric properties comparable to the long form. This instrument could be prospectively used in diverse limb loss populations, such as individuals with upper limb amputation or persons not using a prosthesis. The present results thus crucially contribute to the repertoire of patient-reported outcome measures in the context of post-amputation rehabilitation.

## Introduction

The amputation of a leg requires extensive psychosocial adjustment. Lower limb loss particularly affects the body image, here referring to a component of an individual’s self-concept in terms of perceptions, attitudes, and feelings towards one’s own body, especially its physical appearance^1^. It has been postulated that the body image relies on both sensory and social experiences^2^. As such, the body image of persons with limb amputation can be seen as cognitive-affective response to the loss of the limb^3^, with important social implications: functional or dysfunctional beliefs about the own body and how other people might react to the impairment can affect interpersonal behavior of individuals with limb amputation in terms of the social participation level and the quality of social relationships^2^.

Accordingly, body image disturbances (BID) after amputation have been associated with lower levels of self-esteem (e.g.,^4,5^), quality of life (e.g.,^4–8^), and higher levels of anxiety and depression (e.g.,^4,6,9,10^). Clinically relevant consequences of limb loss in terms of phantom limb pain are positively associated with BID^11^. The extent of BID has been identified as being unique to persons with limb amputation compared to other clinical conditions^12^. Prosthesis use appears to counteract the negative effects of limb loss on the body image. Thus, prosthesis use has been related to reduced levels of physical self-disgust in individuals with lower limb amputation^13^. Likewise, aesthetic and functional prosthesis satisfaction^14,15^ as well as psychosocial and behavioral adaptation to prosthesis use^6,7,10,15^ show beneficial relationships to the body image in persons with limb amputation. These results suggest that restoring the physical integrity of a person with limb amputation by a prosthesis can have positive effects on the user’s bodily self-concept.

Interestingly, no study so far evaluated the association between the body image of a person with limb amputation and prosthesis embodiment, that is, the cognitive integration of a prosthesis into the user’s body representation^16^, although a relationship has been repeatedly assumed^17–19^. Phenomenologically, prosthesis embodiment is expressed in the feeling that the prosthetic device is part of the body rather than a – more or less – helpful tool. Prosthesis embodiment has been identified as major goal of prosthetic rehabilitation^20^ and higher levels of prosthesis embodiment have been associated with higher prosthesis satisfaction and adjustment to prosthesis use^21^. As perceptual correlate of a successful body-prosthesis interaction^16^, an embodied prosthesis restores physical integrity – whose breach is seen as the origin of BID after limb loss. Consistently, the level of prosthesis embodiment can be expected to negatively co-vary with BID.

It can be assumed that the assessment of BID provides crucial diagnostic information regarding the adaption processes after amputation and during prosthetic rehabilitation. The Amputee Body Image Scale (ABIS^4^) was specifically designed to assess BID in persons with lower limb amputation. The ABIS focusses on the individual’s cognitions and attitudes towards the own amputated body and prosthesis use in different (mainly social) contexts. The original English version^4^ has later been psychometrically evaluated and revised in terms of a reduced item number (from 20 to 14) and an alternative response scale^15^. The ABIS has been widely used in international research and has been adapted into several other languages such as French, Portuguese, Turkish, and Chinese^22–25^. Several German translations of the ABIS have been employed^5,26,27^, yet so far, without proper psychometric evaluation. Without a psychometrically sound German ABIS, both research as well as evaluation of rehabilitation activities remain insufficient in German-speaking populations characterized by limb loss. Moreover, since 8 of the original 20 ABIS items focus on the adaptation to prosthesis use, the found relationship between prosthesis adjustment and BID^7,10,15^ could be overestimated. The development of a prosthesis-independent ABIS instrument could thus not only help to evaluate the actual role of prosthetic rehabilitation in research contexts but could also expand its clinical use to individuals who are not using a prosthesis.

Therefore, the present study had three main aims: First, developing and psychometrically evaluating a German adaptation of the ABIS to obtain an instrument that can reliably and validly assess BID in German samples with lower limb amputation. Second, validating the psychometric properties of an ABIS short form from which prosthesis-related items were purposefully removed. And third, evaluating the importance of prosthesis-related variables, including prosthesis embodiment, for the body image in individuals with lower limb amputation, in relation to other variables associated with this outcome.

## Methods

### Recruitment of participants and study setting

Eligible for study participation were individuals who fulfilled the following criteria: unilateral major lower limb amputation (i.e., at least foot level), current prosthesis use, and an age between 18 and 80 years. Participants were recruited using a previously established data base^28^, flyers distributed to rehabilitation centers, and calls utilizing social and print media. A screening interview was conducted over the phone, and if volunteers were found eligible in terms of sufficient skills in the German language, consent forms were sent to them by post or email. As soon as the consent forms were returned, participants received an email with a pseudonymized link to the online questionnaire battery, which was made accessible through Gorilla™ (https://gorilla.sc/); a minority received the questionnaire postally due to having no access to the internet. The battery included items and questionnaires on demographics, amputation, prosthesis use and perceptions, phantom phenomena, and post-amputation pain. Some of these data were already reported in previous reports^16,21,29^. Crucially, ABIS data are reported here for the first time. After on average three months (medium-term stability) and after more than two years (long-term stability), respectively, responses to ABIS items were assessed again, either by sending the questionnaire postally or by email, or filling them in person in the context of on-site experiments. The study was approved by the ethics review board of the Medical Faculty Mannheim, Heidelberg University (reference number: 2016–658 N-MA), and was carried out in accordance with all relevant guidelines and regulations. Written informed consent was obtained from all persons prior to study participation.

### Translation of ABIS items and scale construction

Permission for use and translation of the original English ABIS^4^ was obtained. The translation of the 20 ABIS items was guided by international recommendations for adaptation of self-report measures^30^. Forward translation from English to German was performed by two translators whose native language was German and who were fluent in English. Both translators had a psychological background but only one (the author of the present work) was experienced in post-amputation research. Discrepancies were resolved in a consensus meeting resulting in a synthesis of both forward translations. Back translation of this version was performed by one bilingual person (not two, as recommended) with psychological background but naïve to the study purposes. After another consensus meeting, the pre-final German adaptation of the ABIS items was established. These items were provided to *n* = 14 individuals using a lower limb prosthesis (not included in the main study) who commented on potential difficulties in understanding; these comments were applied if they were deemed as crucial.

Scale construction was guided by previous reports which resulted in the 20-item original ABIS version with a 5-point response scale^4^ (here: ABIS-D) and a 14-item revised version with a 3-point response scale^15^ (here: ABIS-D-R; see also Table 1). However, a crucial restriction of both forms is that its use is limited to (a) individuals with lower limb amputation who (b) use a prosthesis. Particularly the latter feature is essential in the present context, since items focusing on prosthesis perceptions could lead to an overestimation of prosthesis-dependent effects on the body image after lower limb amputation. A site-independent instrument would further enhance comparability and applicability of the instrument in populations with either upper or lower limb amputation. Thus, the author excluded all ABIS-D-R items that are valid only for lower limb amputation (item #13) or involve prosthesis use (items #3, #5, #10, #12, and #14). The remaining eight items (see Table 1) focus on general amputation-related cognitions. The resulting short form (ABIS-D-R-SF) was also used in the following psychometric evaluation and was compared to both the ABIS-D and ABIS-D-R forms.

**Table 1:** Original English and translated German wording of the instruction for the Amputee Body Image Scale (ABIS), its response scales, and items.

	Original English wording	German translation
Instruction	This questionnaire is designed to measure how you see and feel about your body image. It is not a test so there are no right or wrong answers. Please answer each item as carefully and as accurately as you can by placing the appropriate number beside each question.	Die folgenden Fragen sind entwickelt worden, um zu erfassen, welche Gefühle Sie gegenüber Ihrem Körper haben. Es gibt keine richtigen oder falschen Antworten. Bitte geben Sie nachfolgend an, wie oft die jeweilige Aussage auf Sie zutrifft. Wählen Sie jeweils bitte die Antwort, die am besten auf Sie zutrifft.
Response scale	Original: 1 = none of the time 2 = rarely 3 = some of the time 4 = most of the time 5 = all the time Revised: 0 = none of the time 1 = sometimes 2 = most/all of the time	Original: 1 = nie 2 = selten 3 = manchmal 4 = oft 5 = immer Überarbeitet: 0 = nie 1 = manchmal 2 = meistens/immer
**Item 1**	Because I am an amputee, I feel more anxious about my physical appearance in social situations than when I am alone.	Wegen meiner Amputation bin ich in sozialen Situationen besorgter um mein körperliches Erscheinungsbild, als wenn ich allein bin.
Item 2	I avoid wearing shorts in public because my prosthesis would be seen.	Ich vermeide es, in der Öffentlichkeit kurze Hosen zu tragen, da meine Prothese gesehen werden könnte.
Item 3 *	I like my overall physical appearance when wearing my prosthesis.	Ich mag mein allgemeines Erscheinungsbild, wenn ich meine Prothese trage.
**Item 4**	It concerns me that the loss of my limb impairs my body’s functional capabilities in various activities of daily living.	Es beunruhigt mich, dass der Verlust meines Beins die Funktionsfähigkeit meines Körpers bei verschiedenen Aktivitäten des täglichen Lebens beeinträchtigt.
**Item 5**	I avoid looking into a full-length mirror in order *not* to see my prosthesis.	Ich vermeide es, in große Spiegel zu schauen, damit ich meine *Prothese* nicht sehen muss.
**Item 6**	Because I am an amputee, I feel anxious about my physical appearance on a daily basis.	Wegen meiner Amputation bin ich besorgt um mein körperliches Erscheinungsbild.
Item 7	I experience a phantom limb.	Ich nehme ein Phantom meines nicht mehr vorhandenen Beins wahr.
**Item 8**	Since losing my limb, it bothers me that I no longer conform to society’s idea of normal appearance.	Seitdem ich mein Bein verloren habe, stört es mich, nicht mehr das gesellschaftliche Ideal einer normalen körperlichen Erscheinung zu erfüllen.
**Item 9**	It concerns me that the loss of my limb impairs my ability to protect myself from harm.	Es beunruhigt mich, dass der Verlust meines Beins meine Fähigkeit beeinträchtigt, mich vor Schaden zu schützen.
**Item 10**	When I am *not* wearing my prosthesis, I avoid situations where my physical appearance can be evaluated by others (e.g., avoid social situations, swimming pool or beach activities, physical intimacy).	Wenn ich meine Prothese *nicht* trage, vermeide ich Situationen, in denen meine körperliche Erscheinung von anderen bewertet werden könnte (z.B. soziale Situationen, Schwimmbäder, Strandurlaube oder körperliche Intimität).
Item 11	The loss of my limb makes me think of myself as *disabled*.	Durch den Verlust meines Beins betrachte ich mich als behindert.
Item 12 *	I like my physical appearance when *not* wearing my prosthesis.	Ich mag meine körperliche Erscheinung, wenn ich meine Prothese *nicht* trage.
**Item 13**	When I am walking, people notice my limp.	Wenn ich laufe, bemerken andere, dass ich hinke oder humpele.
**Item 14**	When I am wearing my prosthesis, I avoid situations where my physical appearance can be evaluated by others (e.g., avoid any social situations, swimming pool or beach activities, physical intimacy).	Wenn ich meine Prothese trage, vermeide ich Situationen, in denen meine körperliche Erscheinung von anderen bewertet werden könnte (z.B. soziale Situationen, Schwimmbäder, Strandurlaube oder körperliche Intimität).
**Item 15**	People treat me as disabled.	Menschen behandeln mich, als sei ich behindert.
Item 16 *	I like the appearance of my stump anatomy.	Ich mag das Aussehen meines Stumpfs.
Item 17	I wear baggy clothing in an attempt to hide my prosthesis.	Ich trage weite Kleidung, um meine Prothese zu verbergen.
**Item 18**	I feel I must have four normal limbs to be physically attractive.	Ich habe das Gefühl, vier normale Gliedmaßen haben zu müssen, um körperlich attraktiv zu sein.
Item 19	It is important the size of my prosthesis and remaining anatomy of the affected limb are the same size as the other limb.	Es ist wichtig, dass die Größe meiner Prothese und meines Stumpfs mit der Größe meines intakten Beins übereinstimmt.
**Item 20**	I avoid looking into a full-length mirror in order *not* to see my stump anatomy.	Ich vermeide es, in große Spiegel zu schauen, damit ich meinen *Stumpf* nicht sehen muss.

Note: All 20 items constitute the ABIS-D with the original response scale; 14 **bold** items constitute the ABIS-D-R with the revised response scale; and 8 **bold and underlined** items constitute the ABIS-D-R-SF with the revised response scale; * inverted item.

### Other data and instruments

#### Demographic and clinical data

Participants were asked to indicate their sex (male, female, non-binary) and age in years. They were further asked for side (left, right), level (response alternatives: foot amputation; transtibial (lower leg) amputation; knee exarticulation; transfemoral (upper leg) amputation; hip extraction or hemipelvectomy), and reason of amputation (response alternatives: accident; cancer; peripheral vascular disease; injury; infection; congenital limb deficiency; other reasons; multiple responses were allowed), as well as time since amputation (in years). Further, prevalences of current phantom phenomena and post-amputation pain were assessed, by asking for their presence in the last three months, accompanied by a short description each to avoid misunderstanding: phantom limb awareness (that is, the perceived mental image of the missing limb), phantom limb pain, residual limb pain, and other chronic pain. If the presence of other chronic pain was confirmed, participants were asked to select all body parts in pain from a list.

#### Prosthesis Embodiment Scale for Lower Limb Amputees

The Prosthesis Embodiment Scale for Lower Limb Amputees (PEmbS-LLA) bases on previous work on the psychometric nature of embodiment experiences in non-amputated individuals^31^. With ten items, the instrument assesses the phenomenological integration of a prosthesis into the body representation of an individual with limb amputation in terms of ownership (e.g., ‘The prosthesis is my leg.’), perceived physical integrity (e.g., ‘It feels as if I had two legs.’), agency (e.g., ‘I am in control of the prosthesis.’), and anatomical plausibility (e.g., ‘The prosthesis is in the location where I would expect my leg to be, if it was not amputated.’). Participants were asked to indicate their level of agreement to each statement (using a 7-point Likert scale ranging from ‘strongly disagree’ to ‘strongly agree’) while wearing the prosthesis. The PEmbS-LLA shows good reliability and validity^16^ and was recently further validated by using Rasch analysis^32^, resulting in recommendations for an improved scoring algorithm which was applied in the present study (PEmbS-R-LLA). By summing up all items, participants can thus obtain a sum score between 0 and 27, with higher values representing higher prosthesis embodiment. The instrument was recently recommended for research purposes^33^.

#### Trinity Amputation and Prosthesis Experience Scales – Revised

The Trinity Amputation and Prosthesis Experience Scales – Revised (TAPES-R)^34^ are a widely used instrument developed to multidimensionally assess psychosocial adaptation to limb amputation. For the present study, a German translation of the TAPES-R was used (provided by the Center for Orthopedic and Trauma Surgery, Heidelberg University Hospital, Heidelberg, Germany; see also^16,21^). Specifically, the information of two separate parts of the TAPES-R was used: (a) the psychosocial functioning subscales, that is, General Adjustment, Social Adjustment, and Adjustment to Limitation (5 items each, each item was answered using a 4-point Likert scale ranging from 1 ‘strongly disagree’ to 4 ‘strongly agree’, and the average was calculated for each scale, with higher scores being indicative of better general and social adjustment and worse adjustment to limitation, respectively), and (b) the prosthesis satisfaction subscales that measure aesthetic (three items) and functional prosthesis satisfaction (five items; each item was answered using the response scale 1 ‘not satisfied’, 2 ‘satisfied’, and 3 ‘very satisfied’, and the sum score served as measure of feature satisfaction, with higher values representing higher satisfaction).

#### Prosthetic Limb Users Survey of Mobility

The Prosthetic Limb Users Survey of Mobility – 12 item version (PLUS-M-12^35^) assesses a prosthesis user’s perceived ability to perform specific activities, covering movements that range from basic ambulation to complex locomotor activities. Items are scored using five response options that reflect the degree of difficulty in performing each activity, from 1 ‘unable to do’ to 5 ‘without any difficulty’. The questionnaire is scored by summing all item scores, thereby creating a total raw score, which is then converted into T-scores. Using Rasch analysis, the German translation of the PLUS-M-12 has recently been shown to have satisfying psychometric characteristics^36^.

#### General Depression Scale

The Allgemeine Depressions Skala – Kurzform (ADS-K^37^; i.e., General Depression Scale – Short Form) bases on the Center for Epidemiological Studies Depression Scale and is validated for German-speaking populations. The 15 items of the instrument assess depressive symptoms during the last week. Participants were asked to rate statements typical for depression on a scale from 0 ‘rarely’ to 3 ‘most of the time’. The sum score ranges from 0 to 45, with higher values reflecting more severe depressive symptoms.

#### State-Trait Anxiety Inventory

The State-Trait Anxiety Inventory (STAI^38^) is an instrument with 40 items. In the present study, only the trait part was used, consisting of 20 items assessing the frequency of anxious behavior or cognitions that were rated on a 4-point Likert scale ranging from 1 ‘almost never’ to 4 ‘almost always’. The sum score (potentially ranging from 20 to 80) is a measure of trait anxiety, with higher scores reflecting higher anxiety.

### Statistical analysis

All analyses were performed with IBM SPSS v27. Descriptive analyses of all included variables and instruments were performed, and prevalence (%), means (*M*), and/or standard deviations (*SD*) are provided, based on the scaling level. For the ABIS instruments, mean values and floor and ceiling effects are reported. Further, the correlation between the three sum scores was analyzed using Pearson correlations with the co-efficient *r*. Principal component analysis was performed to identify the factor structure underlying all German ABIS versions, based on extracted eigen values and visual inspection of the scree plot. Principal component analysis was conducted with oblique rotation (direct oblimin, delta = 0), and maximum number of iterations was set to 20. Extracted communalities, Kaiser-Meyer-Olkin measure for sampling adequacy as well as for individual items, Bartlett’s test of sphericity, and explained variances are reported. Internal consistency of each ABIS version was evaluated using Cronbach’s alpha, and test-retest reliability (medium-term, long-term) was tested by using intra-class correlation co-efficients. Two-tailed *t* tests were further used to assess stability of the measures, and Cohen’s *d* is provided as effect size. Validity of the instruments was assessed by performing Pearson correlations or *t* tests, respectively, for sex, amputation level, post-amputation pain, psychosocial adaption to amputation and prosthesis use, prosthesis embodiment, mobility, and psychopathology. Note that Bonferroni-corrected *p* values (*p*_Bonf_) are reported whenever multiple testing was applied; non-corrected *p* values are only reported when contextually relevant. In all cases, the significance level is *α* = 0.05. Since the assessed constructs show high interrelationships, multiple regressions on ABIS sum scores as criterion were performed, with entering all variables with significant univariate relationship to the criterion as regressors. For each model, the analysis of variance testing for significance of *R*^*2*^ is reported, along with the adjusted *R*^*2*^ and – for each regressor – the unstandardized coefficient *B*, its standard error *SE*, the standardized regression coefficient *ß*, and the respective *p* value.

## Results

### Sample

In total, the complete ABIS data of *N* = 191 individuals with unilateral lower limb amputation were included (69.1% male, 30.9% female, no non-binary participants). The mean age of participants was *M* = 56.66 years (*SD* = 11.08), with a range from 22 to 80 years. The amputation dated back on average *M* = 27.45 years (*SD* = 16.40), with a range from 0 (that is, the amputation dated back less than 12 months) to 72 years (with one missing value). The majority of participants (61.3%) suffered amputation of the left leg. Most participants reported transtibial (46.6%) or transfemoral amputation (44.0%); only a minority reported foot amputation (2.6%), knee exarticulation (5.8%), or hip extraction or hemipelvectomy (1.0%). Assigning knee exarticulated participants to the above-knee amputation group, there were 50.8% in this category, while 49.2% of participants belonged to the below-knee amputation group. Most participants (64.7%, multiple responses allowed) reported an amputation of traumatic origin (one missing value). Three-month prevalence of phantom limb awareness was 53.4%; prevalence of phantom limb pain was slightly higher (53.9%), emphasizing that phantom limb pain can occur without a clear image of a phantom limb^29^. Prevalence of residual limb pain was 31.4%, and 50.3% reported the presence of other chronic pain, with the neck, shoulders, sacral region, and the remaining leg being the most named body parts (> 30% each). Altogether, 78.5% of participants reported the presence of any pain. Table 2 provides the descriptive statistics for the psychometric instruments used in the present study.

**Table 2:** Descriptive results for the psychometric instruments used in the present study (total *N* = 191 individuals with unilateral lower limb amputation).

Instrument	Missing *n*	Potential range of score	M (SD)
PEmbS-R-LLA	22	0 to 27	18.95 (5.61)
TAPES-R – General Adjustment	2	1 to 4	3.63 (0.48)
TAPES-R – Social adjustment	2	1 to 4	3.45 (0.55)
TAPES-R – Adjustment to limitation	1	4 to 1 *	2.25 (0.79)
TAPES-R – Aesthetic satisfaction	0	3 to 9	6.74 (1.51)
TAPES-R – Functional satisfaction	0	5 to 15	11.38 (2.39)
PLUS-M-12	2	21.8 to 71.4 **	56.03 (9.44)
ADS-K	1	0 to 45	7.63 (7.12)
STAI-T	1	20 to 80	34.32 (10.21)

*M* mean, *SD* standard deviation, *n*  number, PEmbS-R-LLA: Revised Prosthesis Embodiment Scale for Lower Limb Amputees, * TAPES-R* Trinity Amputation and Prosthesis Experience Scales – Revised, * PLUS-M-12* Prosthetic Limb Users Survey of Mobility – 12 item short form, * ADS-K* Allgemeine Depressions Skala – Kurzform [General Depression Scale – short form], * STAI-T* State-Trait Anxiety Inventory – Trait; * note the reversed scoring; ** raw scores (range from 12 to 60) were converted to T scores.

### ABIS translation

No difficulties occurred during the ABIS translation process. Initial testing of ABIS items in *n* = 14 individuals using lower limb prostheses revealed that the translation was well accepted, and no major comprehension issues occurred. The German translation – together with the original English wording – of the ABIS items is provided in Table 1. Average values in the present main study were *M* = 48.52 (*SD* = 14.07) for the ABIS-D (potential range: 20–100), *M* = 11.44 (*SD* = 5.75) for the ABIS-D-R (potential range: 0–28), and *M* = 5.99 (*SD* = 3.54) for the ABIS-D-R-SF (potential range: 0–16), respectively. There were no ceiling or floor effects (i.e., absence of minimal or maximal scores) for the ABIS-D and ABIS-D-R; *n* = 2 participants (i.e., 1.0%) obtained the minimal score in the ABIS-D-R-SF. The ABIS scores showed high correlation with each other (all *r* ≥ .89; all *p*_Bonf_ < 0.001).

### Principal component analysis

Three separate principal component analyses were conducted on the items of the different scales, applying Varimax rotation and Kaiser’s criterion each. Extracted communalities were sufficient for the ABIS-D items (all ≥ 0.41; *M* = 0.60; *SD* = 0.09) and ABIS-D-R items (all ≥ 0.45; *M* = 0.59, *SD* = 0.11) and lower for ABIS-D-R-SF items (all ≥ 0.16; *M* = 0.51, *SD* = 0.17; here, the lowest communality was caused by item #15 ‘People treat me as disabled.’). The Kaiser-Meyer-Olkin (KMO) measure verified sampling adequacy for each analysis, all KMO values ≥ 0.89, and all KMO values for individual items were above the acceptable limit of 0.50. Bartlett’s test of sphericity was highly significant (*X*^*2*^ ≥ 575.55, *p* < .001 each), indicating that correlations between items were sufficiently large for conducting principal component analysis. Four (ABIS-D) and three (ABIS-D-R) components, respectively, had eigen values over Kaiser’s criterion; however, in both scales, component 1 explained considerably more variance compared to the other components (39.5% with an eigen value of 7.90 and 42.5% with an eigen value of 5.95, respectively). Visual inspection of the scree plot further suggests sufficient unidimensionality of both versions. For the ABIS-D-R-SF, statistical support for unidimensionality was even clearer: principal component analysis extracted a single component (eigen value of 4.08) that explained 51.0% of the variance.

### Reliability

Cronbach’s alpha was 0.90 for ABIS-D items, 0.89 for ABIS-D-R items, and 0.86 for ABIS-D-R-SF items, suggesting high internal consistency of the scales. Further, corrected item-scale correlations were > 0.20 in each scale, except for item #7 (‘I experience a phantom limb.’; item-scale correlation of < 0.01) in the ABIS-D. Medium-term stability of the construct was investigated in *n* = 49 participants after *M* = 2.94 months (*SD* = 0.77; range: 2–6 months), and long-term stability was investigated in *n* = 48 participants after *M* = 29.31 months (*SD* = 6.40; range: 16–42 months); overlap of *n* = 19 participants. No instrument showed significant changes from pre to post test-retest interval (scores are provided in Table 3), neither in the medium- nor in the long-term (*t* tests for the difference: all *t*_47-48_ ≤ 1.80, all *p*_Bonf_ ≥ 0.24, all *d* ≤ 0.26). The data thus emphasize that the instrument assesses body image as a stable trait phenomenon. With intraclass correlation co-efficients between 0.86 and 0.95 (medium-term) and 0.78 to 0.87 (long-term), test-retest reliability was good to excellent for each scale^39^.

**Table 3:** Amputee Body Image Scale’s (ABIS) sum scores before (pre) and after (post) a medium-term and a long-term test-retest interval. Note that there were no significant changes from pre to post (all Bonferroni-corrected *p* ≥ .24).

	medium-term stability (*n* = 49)		long-term stability (*n* = 48)	
	pre *M* (*SD*)	post *M* (*SD*)	pre *M* (*SD*)	post *M* (*SD*)
ABIS-D	46.33 (12.47)	47.16 (12.78)	43.92 (12.42)	42.60 (10.99)
ABIS-D-R	11.12 (4.91)	11.41 (5.25)	9.96 (5.21)	9.00 (4.76)
ABIS-D-R-SF	6.06 (3.01)	6.27 (3.09)	4.98 (3.03)	4.77 (3.08)

*M* mean, *n* number, *SD*  standard deviation.

### Validity

Female participants (*M* = 53.76, *SD* = 16.08 for ABIS-D; *M* = 13.61, *SD* = 6.35 for ABIS-D-R; *M* = 7.19, *SD* = 4.04 for ABIS-D-R-SF) had significantly higher levels of BID compared to male participants (*M* = 46.18, *SD* = 12.44 for ABIS-D; *M* = 10.47, *SD* = 5.19 for ABIS-D-R; *M* = 5.45; *SD* = 3.16 for ABIS-D-R-SF; all *t*_90.29-189_ ≥ 2.92, all *p*_Bonf_ ≤ 0.013, all *d* ≥ 0.50). Age and time since amputation did not significantly correlate with BID (all *r* between − 0.01 and 0.17, all *p*_Bonf_ ≥ 0.06). Below-knee amputation (*M* = 46.16, *SD* = 13.99 for ABIS-D; *M* = 10.48, *SD* = 5.60 for ABIS-D-R; *M* = 5.31, *SD* = 3.43 for ABIS-D-R-SF), compared to above-knee amputation (*M* = 50.81, *SD* = 13.84 for ABIS-D; *M* = 12.37, *SD* = 5.77 for ABIS-D-R; *M* = 6.65, *SD* = 3.53 for ABIS-D-R-SF), was associated with significantly lower levels of BID (*t*_189_ ≥ -2.30, significance level between *p* = .023 and *p*_Bonf_ = 0.026, all *d* ≥ − 0.33). Presence vs. absence of post-amputation pain, regardless of the type of pain, significantly predicted BID in all ABIS scales (*M* = 50.63, *SD* = 13.91 vs. *M* = 40.80, *SD* = 11.92 for ABIS-D; *M* = 12.29, *SD* = 5.81 vs. *M* = 8.34, *SD* = 4.32 for ABIS-D-R; *M* = 6.55, *SD* = 3.58 vs. *M* = 3.95, *SD* = 2.54 for ABIS-D-R-SF), all *t*_189_ ≥ -4.05, all *p*_Bonf_ < 0.001, all *d* ≥ − 0.71. This pattern could be observed for any type of post-amputation pain (phantom limb pain, residual limb pain, and other chronic pain; all *t*_189_ ≥ -2.35, all significance levels between *p* = .020 and *p*_Bonf_ = 0.007, all *d* ≥ − 0.34). Presence/absence of PLA, however, was not significantly associated with BID (all *p*_Bonf_ ≥ 0.86, all *d* ≤ − 0.16). Correlations between ABIS measures and the other psychometric instruments are provided in Table 4. All measures showed small to medium relationships in the expected direction, and each correlation remained highly significant even after applying Bonferroni correction (all *p*_Bonf_ < 0.001).

**Table 4:** Relationships between the Amputee Body Image Scale’s (ABIS) sum scores and other psychometric instruments. Note that each correlation was significant (all Bonferroni-corrected *p* < .001).

Psychometric instrument	Pearson correlation co-efficient *r*		
	ABIS-D	ABIS-D-*R*	ABIS-D-*R*-SF
PEmbS-R-LLA	− 0.54	− 0.52	− 0.49
TAPES-R General Adjustment	− 0.48	− 0.50	− 0.54
TAPES-R Social adjustment	− 0.62	− 0.61	− 0.56
TAPES-R Adjustment to limitation	0.54	0.50	0.50
TAPES-R Aesthetic satisfaction	− 0.35	− 0.33	− 0.33
TAPES-R Functional satisfaction	− 0.36	− 0.38	− 0.38
PLUS-M-12	− 0.53	− 0.49	− 0.45
ADS-K	0.44	0.47	0.49
STAI-T	0.46	0.48	0.46

*PEmbS-R-LLA* Revised Prosthesis Embodiment Scale for Lower Limb Amputees,* TAPES-R* Trinity Amputation and Prosthesis Experience Scales – Revised,* PLUS-M-12* Prosthetic Limb Users Survey of Mobility – 12 item short form,* ADS-K* Allgemeine Depressions Skala – Kurzform [General Depression Scale – short form],* STAI-T* State-Trait Anxiety Inventory – Trait.

### Multivariate analysis

In the multiple linear regression analyses on the ABIS sum scores as criterion, all variables with a univariate relationship, as identified in the validity analyses, were entered as regressors. Each model explained 50 to 60% of variance (adjusted *R*^*2*^) which represented a highly significant amount each (all *p* < .001). In all analyses, only prosthesis-related features emerged as significant predictors, with adjustment to limitation, social adjustment to prosthesis use, and prosthesis embodiment being the three regressors which emerged consistently in each model. Statistical details of the analyses are provided in Table 5.

**Table 5:** Regression analyses on body image disturbances (as assessed with the Amputee Body Image Scale, ABIS) with regressors that were identified as being univariately related to the criterion in *n* = 168 individuals with lower limb amputation.

Criterion	Regressors	B	SE	β	*p*	*R* ^2^	adjusted *R*^2^	*p* for *R*^2^
ABIS-D	(constant)	94.51	12.02		< 0.001	0.63	0.60	< 0.001
	Sex ^a^	− 2.06	1.60	− 0.07	0.20			
	Level of amputation ^b^	0.17	1.52	0.01	0.91			
	Post-amputation pain ^c^	1.62	1.79	0.05	0.37			
	**PEmbS-R-LLA**	**− 0.53**	**0.15**	**− 0.21**	**0.001**			
	TAPES-R – General Adjustment	0.27	0.42	0.05	0.52			
	**TAPES-R – Social Adjustment**	**− 1.64**	**0.30**	**− 0.32**	**< 0.001**			
	**TAPES-R – Adjustment to Limitation**	**0.75**	**0.22**	**0.21**	**0.001**			
	**TAPES-R – Aesthetic Satisfaction**	**− 1.16**	**0.52**	**− 0.13**	**0.028**			
	TAPES-R – Functional Satisfaction	0.24	0.36	0.04	0.51			
	**PLUS-M-12**	**− 0.37**	**0.11**	**− 0.24**	**0.001**			
	ADS-K	− 0.02	0.19	− 0.01	0.90			
	STAI-T	0.15	0.13	0.11	0.26			
ABIS-D-R	(constant)	30.21	5.29		< 0.001	0.57	0.54	< 0.001
	Sex ^a^	− 1.07	0.71	− 0.09	0.13			
	Level of amputation ^b^	0.18	0.67	0.02	0.79			
	Post-amputation pain ^c^	0.63	0.79	0.05	0.42			
	**PEmbS-R-LLA**	**− 0.20**	**0.07**	**− 0.20**	**0.003**			
	TAPES-R – General Adjustment	0.02	0.18	0.01	0.90			
	**TAPES-R – Social Adjustment**	**− 0.65**	**0.13**	**− 0.31**	**< 0.001**			
	**TAPES-R – Adjustment to Limitation**	**0.21**	**0.10**	**0.15**	**0.034**			
	TAPES-R – Aesthetic Satisfaction	− 0.30	0.23	− 0.08	0.19			
	TAPES-R – Functional Satisfaction	− 0.04	0.16	− 0.02	0.81			
	**PLUS-M-12**	**− 0.11**	**0.05**	**− 0.17**	**0.019**			
	ADS-K	0.04	0.08	0.05	0.65			
	STAI-T	0.05	0.06	0.10	0.35			
ABIS-D-R-SF	(constant)	17.55	3.34		< 0.001	0.54	0.50	< 0.001
	Sex ^a^	− 0.56	0.44	− 0.08	0.21			
	Level of amputation ^b^	0.40	0.42	0.06	0.34			
	Post-amputation pain ^c^	0.68	0.50	0.08	0.18			
	**PEmbS-R-LLA**	**− 0.09**	**0.04**	**− 0.14**	**0.044**			
	TAPES-R – General adjustment	− 0.17	0.12	− 0.12	0.15			
	**TAPES-R – Social adjustment**	**− 0.31**	**0.08**	− 0.24	**< 0.001**			
	**TAPES-R – Adjustment to limitation**	**0.14**	**0.06**	**0.16**	**0.022**			
	TAPES-R – Aesthetic satisfaction	− 0.24	0.14	− 0.11	0.10			
	TAPES-R – Functional satisfaction	− 0.01	0.10	− 0.01	0.90			
	PLUS-M-12	− 0.05	0.03	− 0.12	0.12			
	ADS-K	0.04	0.05	0.07	0.49			
	STAI-T	0.01	0.04	0.04	0.74			

^a^ 0 = female, 1 = male; ^b^ 0 = below-knee, 1 = above-knee; ^c^ 0 = no, 1 = yes; significant regressors are given in **bold**;* PEmbS-R-LLA* Revised Prosthesis Embodiment Scale for Lower Limb Amputees,* TAPES-R* Trinity Amputation and Prosthesis Experience Scales – Revised,* PLUS-M-12* Prosthetic Limb Users Survey of Mobility – 12 item short form,* ADS-K* Allgemeine Depressions Skala – Kurzform [General Depression Scale – short form],* STAI-T* State-Trait Anxiety Inventory – Trait.

## Discussion

The present study aimed at developing a German adaptation of the Amputee Body Image Scale (ABIS^4^), testing a prosthesis-independent version, and thus quantifying the importance of prosthesis adaptation for a functional body image in individuals with lower limb amputation. Psychometric evaluation of the German ABIS revealed high reliability in terms of internal consistency and long-term test-retest stability as well as meaningful relationships to allied psychological and clinical features. Crucially, the short form of the German ABIS, i.e., the ABIS-D-R-SF, showed comparable or even better psychometric quality compared to the long forms, allowing for usage in diverse populations characterized by limb loss. The present results showed specific inverse relationships between BID and prosthesis-related factors in terms of psychosocial adjustment and prosthesis embodiment, emphasizing the important role prosthetic rehabilitation plays for mental well-being. Thus, the results are not only relevant for German-speaking populations characterized by limb amputation but have implications for international stakeholders in rehabilitation and rehabilitation research.

The German adaptation of the ABIS showed good to excellent psychometric properties, and thus ranks this instrument into a growing number of translated ABIS versions^22–25^. However, some features of the original ABIS can be objected. First, its use is limited to individuals who use a prosthesis. One can rightly counter that most individuals with lower limb amputation living in Germany use a prosthesis^40^, but it would still be desirable to have an instrument that dispenses with this limitation. Second, the ABIS is only applicable in individuals with lower limb amputation since some items are valid only for the leg. However, there is reason to assume that individuals with upper limb amputation pass through similar changes in their body image, and there is preliminary evidence that they even suffer more compared to persons with lower limb amputation^41^. Without an appropriate tool that is independent of the type of limb loss and prosthesis use, crucial research questions remain unanswered. The short version of the ABIS proposed in the present study could close this critical gap, given that its psychometric properties are comparable or even superior to the long forms. In prospective studies, the instrument could easily be adapted to other populations, by exchanging the word ‘leg’ by ‘arm’ or using the neutral term ‘limb’. The present instrument could thus make a crucial contribution to the monitoring of rehabilitation outcomes in clinical practice and research. However, it must be clearly emphasized that validation of the ABIS short form in diverse limb loss populations, as well as its translation into other languages, is still pending. Therefore, the present results on the ABIS short form must be viewed as preliminary.

The temporal stability of the body image in the present study is remarkable. While previous ABIS studies evaluated construct stability over short time periods of between two and seven days^22,24,25^, the current results indicate long-term stability of up to two and a half years. Previous cross-sectional studies comparing different age cohorts also postulated high stability of the body image in non-clinical populations (e.g.,^42,43^). However, long-term stability of the body image in clinical populations in general, and in individuals with limb amputation in particular, has only been studied sparsely. The present data suggest that the body image in individuals with limb amputation represents a stable cognitive trait. Since there was no significant relationship to the time since amputation, this suggests that the body image may manifest soon after limb amputation. However, it remains open whether rehabilitation experiences or psychological adjustment can positively affect BID once manifested. During the review process for this article, the question arose as to whether events were recorded that occurred during the test-retest interval and that potentially influenced the participants’ response behavior. Unfortunately, this information is not available; the absence of any significant differences between pre- and post-measurements, however, supports the conclusion of temporally stable body-related cognitions after limb amputation which are not affected by unspecific factors (e.g., memory capacity) potentially associated with shorter test-retest intervals. Longitudinal studies covering a time frame from before to after surgical limb amputation or before and after the implementation of a new rehabilitation activity might further help to identify temporal dynamics of the body image as well as critical time windows. In the context of eating disorders, previous results already showed positive effects of a successful treatment on body dissatisfaction (e.g.,^44^), suggesting that tailored interventions might also be effective for individuals with limb amputation characterized by perpetuated BID.

A positive body image was consistently linked to psychosocial adjustment to the amputation and prosthesis use in the multivariate analyses. Interestingly, this relationship absorbed the significant univariate effects of other variables, such as sex, level of amputation, post-amputation pain, or psychopathology. This is important methodological information for prospective studies, since the high level of interrelationships between different factors bears the risk of misjudging the importance of a single factor. The present results suggest that prosthetic rehabilitation plays a major role for the establishment of a healthy physical self-concept after lower limb amputation. Crucially, the present study provided first-time evidence for a specific relationship between prosthesis embodiment and a beneficial body image in individuals with lower limb amputation. Prosthesis embodiment as assessed in the present study refers to a basic perceptual phenomenon resulting from the integration of bottom-up sensorimotor information (e.g.,^45^); the inverse correlation to BID emphasizes that also higher-order body cognitions rely – at least partly – on these fundamental sensorimotor inputs. The present findings thus join a growing number of positive rehabilitative outcomes associated with prosthesis embodiment in individuals with lower limb amputation, such as mobility, prosthesis satisfaction, or reduced levels of post-amputation pain^21,46^. This emphasizes that the restoration of perceived physical integrity should represent a major rehabilitative goal in individuals with lower limb amputation, with the same relevance as functional or aesthetic restoration.

Social participation has been identified as crucial for the rehabilitation of individuals with limb amputation^47^. Accordingly, social comfort is related to higher levels of mental well-being in this population^48,49^. Murray^50,51^ emphasized the social meaning of prosthesis use and concluded that using a prosthetic device reduces social stigmatization and thus facilitates social integration after limb amputation. The body image crucially relies on social expectations^2^ which may explain the strong inverse relationships between BID and psychosocial adjustment to the amputation and prosthesis use in the present study. This is in line with previous results showing that the subjective level of prosthesis acceptance by relatives is closely related to a healthy body image in individuals with lower limb amputation^22^. Interestingly, the present data also indicate that prosthesis embodiment has social implications, since its presence might reduce the anticipation of (actual or expected) negative social reactions. This is an important conclusion which needs further attention in prospective studies. Experimental data in non-amputated participants already showed that basic body perceptions modulate social cognitions^52^, potentially giving prosthesis embodiment a new social relevance in the rehabilitation process after lower limb amputation.

Several limitations of the present study must be noted. First, the sample is composed of rather well-adapted individuals, with on average more than 25 years of experience with limb loss and prosthesis use. This limits the generalizability of conclusions, whose validity for poorly adapted and/or shortly amputated populations remains open. Although the author recommends using the short form of the ABIS for prospective studies focusing on BID in diverse populations with limb loss, the instrument is not validated for such an extended use. Without studies on appropriate comparative samples – such as individuals with congenital limb loss, upper limb amputation, or without prosthesis use –, the use in these populations must be considered exploratory. Future studies should also focus on mechanistic and causal links between psychosocial adaptation to prosthesis use and the body image. So far, all links reported in the present study are of correlative nature, so that the directionality of relationships cannot be determined with certainty: psychosocial adaptation could cause a better body image, but a better body image could also lead to higher levels of psychosocial adaptation. Prospective experimental studies are needed to answer this critical question. These studies could further aim at the underlying technical features. For example, a recent study showed that microprocessor-controlled prostheses, compared to mechanical ones, are associated with a more beneficial body image^53^. Sensory feedback has also been proposed to positively affect the prosthesis user’s body image^54^. In turn, advanced prostheses facilitate the emergence of prosthesis embodiment^46,55^. Identifying the potentially mechanistic relationships of these effects in prospective studies might give important impulses to both prosthesis developers as well as clinicians implementing rehabilitation activities for individuals with lower limb amputation.

## Conclusion

With the German translation of the ABIS, the present study provides a psychometrically sound instrument for the assessment of BID in German-speaking populations characterized by limb loss. The results thus crucially contribute to the repertoire of patient-reported outcome measures in the context of post-amputation rehabilitation in German-speaking countries. However, the findings have significance beyond a use in German cohorts: the multivariate analyses revealed a specific relationship between BID and psychosocial adaptation to the amputation and prosthesis use. Particularly the inverse association between BID and prosthesis embodiment has theoretical and practical relevance, with the potential to impact future prosthesis development. By allowing a prosthesis-independent assessment of BID, the ABIS short form could further fill a critical gap in rehabilitation research and clinical practice. The need for such an instrument was recently postulated in a review on the assessment of the body image in individuals with lower limb loss^56^. However, future studies have yet to demonstrate the psychometric sufficiency in diverse populations; until then, the results regarding the ABIS-D-R-SF have to be considered preliminary.

## Data Availability

The dataset analyzed for the current study is available from the author on reasonable request.
